# Factors Associated with Chronic Kidney Disease of Unknown Etiology (CKDu): A Systematic Review

**DOI:** 10.3390/healthcare11040551

**Published:** 2023-02-13

**Authors:** Swetalina Nayak, Tanveer Rehman, Kripalini Patel, Pujarini Dash, Alice Alice, Srikanta Kanungo, Subrata Kumar Palo, Sanghamitra Pati

**Affiliations:** ICMR-Regional Medical Research Center, Bhubaneswar 751023, India

**Keywords:** chronic kidney disease of unknown etiology, associated factors, systematic review, risk factors, farming, water sources

## Abstract

(1) Background: Despite ample research, the factors, specific causes, and pathways associated with chronic kidney disease of unknown etiology (CKDu) remain elusive. Therefore, we performed a systematic review to explore the potential etiologies for the development of CKDu globally. (2) Methods: A systematic literature review was conducted using databases CINAHL, Cochrane Library, Embase, Google Scholar, MEDLINE, and PsycINFO on the specific causes and pathophysiology related to CKDu from inception until April 2021. Study selection, data extraction of included articles, and quality appraisal were assessed. The narrative approach was used to summarize and comprehend the findings. (3) Results: Our study included 25 studies, considering 38,351 participants. Twelve studies were case–control, ten were cross-sectional, and three were cohort designs. All articles were from low-and middle-income countries (LMICs). The findings suggest 12 factors are associated with CKDu. Most studies (*n* = 8) identified farming and water sources as the factors related to CKDu, with heavy metal toxicity coming in second (*n* = 7). (4) Conclusion: The systematic review reported various factors associated with CKDu, from which most studies reported farming, water sources, and heavy metal poisoning. Considering the findings, the study recommends future strategies and public health initiatives to prevent the epidemiological/environmental factors contributing to CKDu.

## 1. Introduction

Noncommunicable diseases (NCDs) have become a major global health challenge due to the rapid epidemiological transition [1]. In 2017, deaths attributable to NCDs accounted for 73% of the total global deaths [2]. The distribution of NCDs was most prominent in high-income countries (HICs) in the early years. However, it has rampantly increased in low-and-middle-income countries (LMICs) in recent years, contributing to a rise in NCD-related morbidity and mortality [3].

One key determinant of NCDs’ poor health outcomes is chronic kidney disease (CKD) [4]. According to the Global Burden of Disease Study 2017, the global prevalence of CKD was 9.1% (697.5 million cases), and 1.2 million deaths were attributed to it, resulting in CKD being the 12th leading cause of death worldwide [5,6]. In addition, there is evidence to indicate a multifactorial role in the progression toward kidney failure, but not all causes have been investigated so far [7]. 

Epidemiological studies conducted in HICs have found some known traditional risk factors for the progression of kidney disease, such as ageing, obesity, hypertension, and diabetes [7]. Non-traditional risk factors (such as environmental, occupational, and infectious diseases), disproportionately higher in LMICs, remain unexplored. Hence, kidney diseases without identifiable traditional risk factors are termed CKD of unknown etiology (CKDu) [7,8,9]. However, this definition is not generally agreed upon either. The definition of CKDu has been the subject of numerous discussions in recent years. For instance, many authors limit CKDu to a particular type of CKD frequently found in rural settings in non-diabetic people, accompanied by low-grade proteinuria, and a rapid transition to end-stage kidney disease (ESKD). Many attributed it to farming, cadmium exposure, heat exposure, lack of fresh water, and climate change [10]. Ochratoxin A (OTA), a mycotoxin found in numerous food items, was found to cause CKD at even low concentrations [10,11]. Some authors also link CKDu with snake bite and herbal use, frequently reported in South Asia and Central America. Additionally, air pollution, particularly particles with a diameter <2.5 μm, has also been associated with CKD [12,13].The definition varies depending on the region too. Therefore, to avoid confusion, we restrict our definition only by excluding the traditional risk factor of CKD, i.e., diabetes, hypertension, and HIV.

The atypical presentation of CKDu has become epidemic in different regions of the world, which usually remains undiagnosed until its late stage [9]. Over the past few decades, parts of Central America, South Asia, Eastern Europe, and other countries such as Sudan, Tunisia, Tanzania, and the El-Minia Governorate in Egypt reported a similar disease pattern [14]. However, the exact cause remains unidentified to date. Subsequently, a similar endemic was also reported, such as Balkan endemic nephropathy, Mesoamerican nephropathy, and Chinese herb nephropathy, which were named after the area of origin [8,9].

Furthermore, several research studies have postulated possible risk factors for CKDu. For instance, a cross-sectional survey in Nicaragua by Lebov et al. found gender, age, residence, alcohol consumption, higher duration of working in agricultural lands, and having co-morbidities to be positively associated with CKDu [15]. Another cross-sectional study conducted in Sri Lanka by Jayatilake et al. mentioned exposure to heavy metals and pesticides is linked to CKDu [16]. So, several studies have come up with different hypotheses, although the actual cause(s) remains a mystery. Therefore, this systematic review has been conducted to find the contributory factor(s) for the development of CKDu across the globe.

## 2. Materials and Methods

This systematic review is registered with the Prospective Register of Systematic Reviews (PROSPERO) (registration no: CRD42021246096) and has been reported using the Preferred Reporting Items for Systematic Review and Meta-analysis (PRISMA) guidelines [17].

### 2.1. Search Strategy

Using CINAHL, Embase, MEDLINE, and PsycINFO electronic databases, we performed a search to reach out to the peer-reviewed articles published from inception till April 2021. Additional studies that investigated the risk factors for CKDu were explored by searching articles from references of the included articles. Three primary concepts were encapsulated in search terms: CKDu, etiology, and risk factors. The detailed search strategy is provided in Table S1.

### 2.2. Inclusion/ Exclusion Criteria

Articles on original primary research (including observational and experimental studies) published in English and peer-reviewed journals were included. No exclusion criteria were set on the definition of CKDu; anyone diagnosed as CKDu by a doctor or hospital would be included in our study. However, review articles, project reports, expert opinions, narrative reviews, commentaries, case reports, conference papers, and policy analyses were excluded. Furthermore, we excluded studies reporting diabetes and hypertension as associated factors, as these factors were considered the traditional causes of CKD [7].

### 2.3. Study Selection

The electronic and bibliographic searches were conducted independently by two authors (SN and TR), and a third author (KP) removed all the duplicates. In the next stage, studies identified during the search process were imported to a free web-based tool for expediting the primary screening of abstracts and titles [18]. Two authors (AA and SN) analyzed the title, abstract, and keywords against the inclusion criteria, and articles that either reviewer approved progressed to second-stage screening. Two authors (SN and TR) independently reviewed the full-text articles. Any disagreements between the authors were resolved in concordance with the authors having expertise in systematic reviews (SK and SKP). Researchers selected the studies based on the research question: “Does the study identify any potential risk factors associated with CKDu?”.

### 2.4. Quality Assessment

We used the Joanna Briggs Institute (JBI) Critical Appraisal Checklists to assess the quality of the selected articles [19]. In the evaluation, scores for each option were determined mutually by the authors (yes/not applicable = 1, no/unclear = 0).Summation was carried out by taking all scores given to each article. Then the scores assigned to each question were converted into percentages using the total questions. Like this, a numeric quality score ranging from 10 (lowest) to 100 (highest) was given to each article. Studies scoring >70 percent were considered high quality, while articles scoring between 40–70 percent and <40 percent were considered moderate and low, respectively (Table S2). We excluded the articles that were assessed to be of low quality. Two authors (KP and SN) independently evaluated for sources of bias, and the opinion of a third author (TR) was sought if the authors disagreed. 

### 2.5. Data Extraction and Synthesis

Two authors (BN and KP) collected the data independently using a pre-designed table. Another author (TR) cross-checked the data extraction, and any error was corrected through a collaborative assessment of the original publications. The relevant data from the finalized articles were extracted into MS Excel against the following components: 1. study details: author/s, year of publication, and country; 2. study setting; 3. study design; 4. sample size; 5. participants; 6. exposure(s); 7. CKDu prevalence and tool to diagnose it. A meta-analysis was planned initially, but insufficient data were available to pool the results. Hence, the findings were summarised in narrative form after categorizing the articles based on exposure of interest or risk factor(s) for CKDu.

## 3. Results

**Figure 1:**
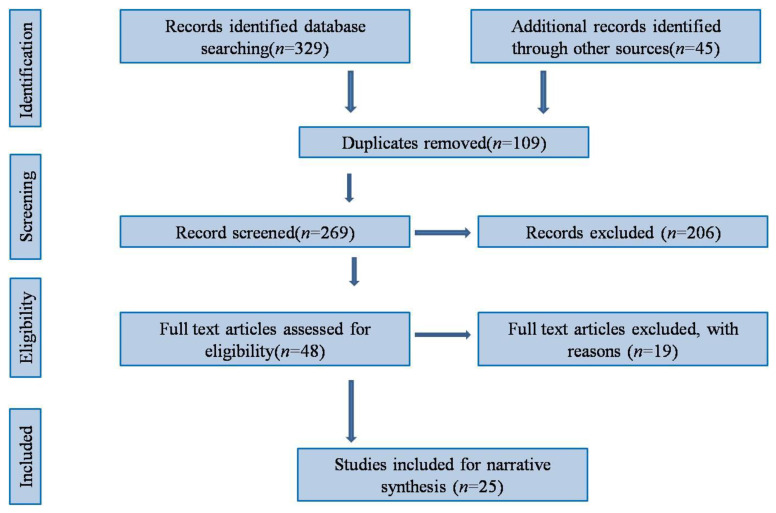
PRISMA flow diagram of the study selection process.

After an extensive literature search, we retrieved a total of 329 articles, the majority (71.7%) of which were extracted from the MEDLINE database. After removing the duplicates, 269 articles were screened for title and abstract, and 48 relevant articles were considered for eligibility. After the full-text evaluation and quality assessment, 25 articles that qualified for the required criteria were finally included. Figure 1 details the PRISMA flow diagram of the study selection process. The list of excluded articles is provided in Table S3. Additionally, the PRISMA checklist is provided in Table S4.

### 3.1. Characteristics of the Studies

This review covered publications from seven countries, all of which were from LMICs. Among the 25 articles included, 16 were from Sri Lanka, followed by two from India, Mexico, and El Salvador, and one from China and Malawi. All studies were conducted between the years 2000 and 2021. Of the total, 12 were case–control studies, followed by ten cross-sectional design studies and three cohort study designs. The included studies represent 38,351 participants, with the sample size ranging from 46 to 24,726. The details about the included studies are provided in Table 1, Table 2, Table 3 and Table 4. A total of 17 studies were identified as mentioning their male and female status. In this, a total of 1933 males and 1368 females were reported. Seven studies were hospital based, whereas 22 were community-based.

### 3.2. Narrative Synthesis

**Figure 2:**
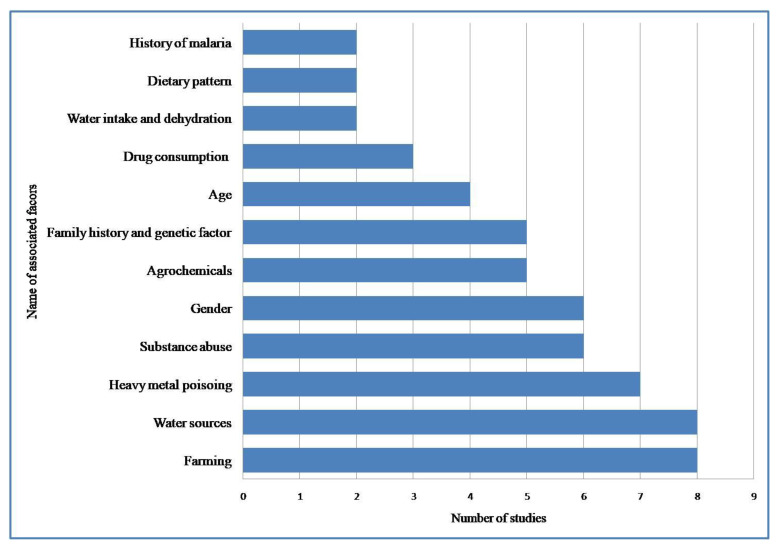
Number of included studies reporting associated factors.

We did not conduct a meta-analysis due to the heterogeneity of method sand lack of information in included studies. Additionally, differing units of measure and variables did not allow for meaningful comparisons. Only simple and descriptive comparisons were reported. We have grouped the factors linked with CKDu into distinct categories: farming, substance abuse (smoking and alcohol consumption), dehydration (water intake and heat stress), heavy metal poisoning, water sources, demographic factors, genetic factors, and miscellaneous. Each of these factors is discussed in detail in the following sections. A graphical depiction focusing on the number of studies reporting elements that could be interpreted as either risk or resilience is given in Figure 2.

## 4. Associated Factors

### 4.1. Farming

A total of eight studies identified farming as a risk factor for CKDu [16,20,21,22,23,24,25,26]. Of these, most of the studies were conducted in Sri Lanka [20,24,25,26], which revealed that agriculture was significantly linked to CKDu compared to other occupations. Upon comparing the results, we found two community-based studies with a mean participant age of 47.8 years (SD = 9.6years) and 46.6 years (SD = 9.0 years) [24,25] were more likely to be affected in CKDu than the study with mean participant age below 40 years [16]. In contrast, compared to other jobs with a female predominance, a cross-sectional study in a rural location with a mean participant age of 33.5 years (SD = 12.7 years) found farming to be a protective factor (61%) [22].

Across all types of farming, similar conclusions were found. It revealed a positive association between CKDu and banana farming, with the frequency increasing by 77% compared to people in other occupations [23]. In addition, people who live near sugar cane agriculture field areas were significantly associated with developing CKDu compared to those living in different regions [21]. Supporting the assertion mentioned above, one hospital-based study from El Salvador, with a mean participant age of 45, found that sugarcane workers developed severe interstitial fibrosis and tubular atrophy earlier than non-sugarcane agricultural workers [26]. Participants with a mean age of 23.9 years (SD = 3.7 years) and a range of 18–30 years involved in sugarcane farming, such as seeders and cane cutters, had higher odds of the rapidly declining estimated glomerular filtration rate (eGFR) compared to people who did not have a history of working in sugarcane farming [23]. Paddy cultivation, on the other hand, did not appear to be linked to CKDu [16,24].

A total of five studies identified a link between CKDu and agrochemicals [20,22,23,24,27]. Two studies of 41 and 82 participants, of whom the majority of the participants were male with a mean age 47.8 years (SD = 9.6 years) and 45.51years (SD = 19.78 years), respectively, with information on the usage of agrochemicals, found a strong association for CKDu [20,22], compared to a study of mean participant age was 23.9 (SD = 3.7 years) [23]. Similarly, using fertilizers and pesticides such as organophosphate, paraquat, MCPA, Glyphosate, Bispyribac, Carbofuran, and Mancozeb was linked to the development of CKDu [20]. Another study confirmed the findings, demonstrating that over 90% of CKDu patients use agrochemicals in their farming [27].

### 4.2. Water Sources

A total of eight studies revealed a relationship between CKDu with water sources [20,23,24,27,28,29,30,31]. Drinking water from abandoned sources has been a crucial factor for CKDu. According to studies from Sri Lanka, abandoned water sources in endemic areas contained heavy metals (such as calcium (Ca), magnesium (Mg), barium (Ba), strontium (Sr), iron (Fe), titanium (Ti), vanadium (V), lead (Pb), and arsenic (As), sulfate which is 4.5 times higher than non-endemic areas), total dissolved solids, and silica (115.5 mg/L-1) (which is 8 and 12 times higher than in non-endemic area water sources [20,28,29]. Additionally, water analysis from such sources revealed a significantly higher amount of hardness, electrical conductivity, and Glyphosate level. Additionally, consuming water from such sources substantially increases the risk of acquiring CKDu [20,28,29]. Studies also revealed that these total dissolved solids (r value = 0.271) and arsenic (r value = 0.304) also significantly correlated with creatinine levels (*p*-value 0.05). However, phosphate content was inversely correlated with increasing creatinine levels (r value= −0.628, *p*-value 0.001) among CKDu patients [28]. Additionally, the groundwater of endemic areas was acidic (Ph: 5.6). It showed some seasonal variation [29], and most people from the CKDu endemic region drank water from such stagnant irrigated water sources [30]. In contrast to this, another study from a CKDu endemic zone found low levels of trace elements such as cadmium (Cd), arsenic (As), lead (Pb), and uranium (U) in the available water sources [32]. On the other hand, it was also found that people ranging from 18 to 30 years of age who were drinking water from the pipe supply were protected from CKDu development [24].

## 5. Miscellaneous

A total of six studies reported substance abuse [16,20,21,25,33,34]. Alcohol intake and smoking were more related to CKDu than non-alcoholics and non-smokers, respectively [16,20,23,25,33,34]. Though smoking was associated with CKDu in three studies, two showed a non-significant association (*p* value ≥ 0.05). Similarly, tobacco chewing was strongly linked with CKDu, as was solely betel chewing in one study, while in two studies, the same finding did not occur [20].

Two studies looked at water intake and dehydration [23,24]. Dehydration due to heat exposure and insufficient water consumption was a risk factor for CKDu. Working under the sun for more than six hours per day and consuming less than three liters of water per day were found to have a significant risk of acquiring CKDu [24]. Furthermore, the availability of shade during working hours protected against CKDu compared to the unavailability of shade in the workplace [23]. In contrast, a study from Nicaragua with a mean age of 23.9 (SD = 3.7years) indicated that exposure to high temperatures and frequently working in a hot environment had a lower risk of developing CKDu, compared to those who work sporadically in a hot setting [23].

Heavy metal poisoning is a concern for CKDu, and seven studies reported the relationship of CKDu with heavy metal poisoning [29,31,32,35,36,37,38]. Urine beta-2 microglobulin (B_2_M), which is a known indicator of heavy metal poisoning of cadmium, arsenic, and lead [31], alpha1-microglobulin (A1M), and N-acetyl-B-D-glucosaminidase (NAG) are two sensitive markers for cadmium exposure [37]. While NAG excretion was only revealed to be notable in stage 5 of CKDu, it was found that A1M level steadily increased only with early stages of CKDu, compared to their unaffected relatives (control) [37]. In Medawachchiya, the CKDu patients had urine B_2_M levels seven times greater than non-CKDu participants (*p* value < 0.005) [31]. Further, A1M excretion was significantly higher in CKDu patients and steadily increased as the stage increased [36]. Similarly, around 68% of CKDu patients and 28% of the control group had an arsenic level of more than 21 micrograms/gram. The same study revealed that around 48% and 28% of the subjects from cases and control groups, respectively, were diagnosed with chronic arsenic toxicity, indicating a possible link [38]. Additionally, CKDu-affected regions had moderate to high fluoride levels in their water bodies [34]. Further, a study carried out in Sri Lanka showed a higher mean blood lead level (1.48 µM-mean) in 78.9% of CKDu-affected individuals compared to unaffected individuals (0.025 µM) [29].

Two studies found information about dietary patterns [24,31]. Based on the 24-h dietary recall method, considering bread and wheat flour products as the reference food, the consumption of locally produced rice and rice products was significantly higher in CKDu endemic areas than in non-endemic areas. Further, people who consume low-millet products and eat meat regularly have greater attributes than those who eat more millet and do not eat meat [24,31].

Three studies revealed the relationship between drug consumption and CKDu [20,23,34]. According to a Sri Lankan study, 29.7% of CKDu patients consumed nephrotoxic drugs [34]. Investigations also revealed a link between the consumption of NSAID (non-steroidal anti-inflammatory drugs) drugs and CKDu [23]. However, long-term painkiller use was not a significant risk factor for CKDu [20]. Two research studies found an association between the history of malaria and CKDu, which showed a positive association with CKDu [24,25]. Four studies reported that with every 10-yearincrease in age, the chance of occurrence of CKDu increased [16,33,34,38].According to studies, and age of over 40 years was more associated with CKDu [33,34,38]. Similarly, another study found that more than 92% of CKDu patients were more than 39 years of age [16]. Studies also showed the male gender as a predisposing factor for CKDu [16,20,36,38,39]. Studies showed males are more affected than their female counterparts [20,36,38]. Male wage labourers were more likely to have CKDu than their female counterparts [39]. In contrast, another study did not report any association between males and CKDu [16].

A study from Sri Lanka found that a family history of CKD was present among 35.8% of CKDu patients [40]. Research has demonstrated that genetic factors (i.e.,CYP1A1 polymorphism, GSTM1) are associated with a 1.4-2 fold increased risk for CKDu [41,42]. Similarly, another study carried out in Sri Lanka found serum creatinine was positively correlated with two other genes named KIM1and IGFBP1, whereas FN1 and IGFBP3 were negatively related to serum creatinine levels. Again, it revealed that KIM1, IGFBP1, and KLK1 were negatively correlated with eGFR [43]. Another study showed that 35.8% of total CKDu patients had a prior family history of CKDu, which supports the above findings [36]. Only one case–control study from Sri Lanka found snake bites as an association factor for CKDu (26).

## 6. Discussion

CKDu is an emerging public health issue with increased cases reported globally. Because of the absence of any significant clinical signs and symptoms, most cases are reported late, making their management difficult. Identification of its risk factors would help to prevent this disease better. Therefore, this systematic review attempts to identify the established risk factors aiding in the development of CKDu. Our research uncovered 25 publications for consideration, with most studies reporting a link between farming and CKDu. However, other factors were also considered and described.

People working in agriculture or farming carry a potential risk for CKDu. While most research concluded that agriculture and CKDu are related, several studies did not reach the same conclusion. It is interesting to note that neither the quantity of time spent working on sugarcane nor the amount of agricultural activity was related to the final results [23]. Exposure to heat and low water intake causes dehydration, affecting the kidney [32]. Agrochemical usage in agriculture has long been a known risk factor for CKDu [38]. Commonly used agrochemicals such as Glyphosate [27], organochlorine compounds [14], and parquet is extensively used worldwide. Studies have reported a significant association between CKDu and agrochemical use [20,23,25]. “Pesticides” and “herbicides” contain hundreds of toxins with distinct toxicological actions [44], and their use varies widely across crops, regions, and time. Exposure to these chemicals during mixing and application, storage, and disposal carries the risk.

More than half of the population depends on agriculture for livelihood [14]. In this context, educating people in this occupation about the dangers of agrochemicals and how to prevent their exposure is critically important. It is also important to build strategies/policies for their lesser use and advocate preventing further disclosure. Eating food from an endemic region increases the chances of CKDu, which may be attributed to the presence of OTA, the most common contaminant in food and feed, which causes nephrotoxicity. Evidence showed that it also increases renal fibrosis by activating TGF-B1 (transforming growth factor beta one)/SMAD_2/3_ [10,11]. Additionally, snake bite is also found to be linked with CKDu. The pathogenesis of kidney damage caused by snake envenomation includes direct venom cytotoxicity, systemic myotoxicity (rhabdomyolysis), accumulation of large amounts of myoglobin, and ischemia (brought on by systemic bleeding and vascular leakage) [45].

Contamination of drinking water with heavy metals (Ca, Mg, Ba, Sr, Fe, Ti, V, Cd, As, Pb, U) has increased the risk of CKDu [27,40]. Additionally, studies showed that fluoride and Glyphosate in the water source increase the risk of CKDu [14,20]. Based on the findings, it is crucial to ensure safe drinking water to prevent CKDu. Most people drink untreated water, especially those living in rural and remote areas [46,47]. A safe drinking water source or facility is unavailable in many places. The condition worsens during the summer and rainy seasons. It is essential to educate people about the importance of safe drinking water and how they can treat the water they use. However, removing heavy metals, fluoride, and Glyphosate is impossible at the individual level. Initiatives need to be undertaken by government departments to identify the sources of safe drinking water and ensure its availability to people.

While some studies have attributed high temperatures during summer to CKDu, some have attributed low water intake [23,24]. Both conditions lead to dehydration which is the factor for kidney damage leading to CKDu. Excessive sweating brought on by prolonged exposure to heat results in a drop in extracellular fluid, which causes an increase in vasopressin and decreases the glomerular filtration rate. If vasopressin secretion is prolonged, it will destroy the tubulointerstitial tissue and lead to CKD. Furthermore, Roncal-Jimenez et al. hypothesized that elevations in urine osmolality brought on by dehydration activate the aldose reductase pathway, converting glucose to fructose. Fructokinase breaks down fructose in the proximal tubules to produce urate, oxidants, and inflammatory mediators that cause tubular damage [48]. Although the majority of studies established this link, others do not. Some of the data on heat stress were self-reported, and some studies reported that the instrument they used to measure the situation might not be reliable in that population or would not accurately capture cumulative heat stress over time.

Additionally, it was noted that occupational exposures that are not related to heat might endorse the advancement of CKDu [23]. A study supports this by finding that higher water intake is a protective factor for kidney-related problems [49]. Farmers and daily wage labourers are mostly exposed to heat for a prolonged period because of their occupation. This makes them vulnerable to CKDu. Efforts are required to provide shade during hot hours. Proper planning to avoid exposure to hot sunlight could help in preventing CKDu. In addition, we found alcohol as a risk factor for CKDu. Because of the diuretic property of alcohol [50], it also increases dehydration. However, the association of alcohol with CKDu because of its chemical property or diuretic action needs to be explored.

An increase in age, male gender, and family history of CKD were risk factors for CKDu [20,33,34,38,49]. With increased age, cumulative exposure to risk factors increases. However, the study also shows that people in the younger generation are also acquiring CKDu [16]. According to a Sri Lankan study, males were more affected than females [20,39]. Male occupations are chiefly with outdoor activities and are more exposed to risk factors than females, so the prevalence among them is possibly more. However, whether any hormonal protection role is present among females needs to be studied. The genetic relationship for CKDu has been reported, and studies have found a ‘family history of CKD’ as a risk factor for CKDu [40]. The family members of diagnosed cases must be regularly screened and advised for proper care to prevent and manage CKDu.

We discovered several putative CKD risk variables that vary by location, but the variability in their reported relationships with CKD makes it challenging to conclude CKDu etiologies. Asia’s most commonly examined factors were farming, agrochemical use, and water sources, but altitude and temperature were only addressed in America. There are, nevertheless, numerous parallels. Additionally, heavy metals, heat stress, and dehydration, and food exposures were all mentioned in research from all over the world, as were family history, temperature, altitude, and dietary direction.

CKDu is currently a cause for concern, particularly in therapeutic management. In small kidney tumors in patients, particularly in weaker ones, percutaneous cryoablations (PCA) and percutaneous thermal ablation are playing an increasingly significant role [51]. While microwave ablation is still regarded as experimental due to its more recent use and a weaker body of facts, radiofrequency ablation is currently considered an established procedure by the American Urological Association (AUA) and the European Association of Urology [52]. Regardless of the surgical technique that doctors are most comfortable with, most urological recommendations advocate partial nephrotomy as the first-line treatment option for renal masses when surgery is necessary [53,54]. Furthermore, 3D guiding for nephron-sparing surgery is linked to lower kidney injury and damage rates. Notably, less non-tumor renal parenchyma is sacrificed or exposed to ischemia after resection, and the opening of the collecting system occurs less frequently. Utilizing 3D technology, however, has not been shown to significantly improve oncological or functional outcomes.

The study has some limitations. A meta-analysis was not possible because of the diversity of the outcomes assessed. We could not statistically evaluate the effects of the result reporting bias for the same reason. This study only looked at studies about CKDu, and the most well-known risk factors for CKD, such as hypertension and diabetes, were not considered. It is also likely that we overlooked some critical aspects due to our inclusion criteria.

## 7. Conclusions

Multiple risk factors have been reported to associate with CKDu. While factors such as many modifiable environmental and personal factors largely determine the disease’s occurrence. Exposure to agrochemicals, pesticides, herbicides, heat, alcohol, and tobacco could be addressed by creating awareness among people at risk. In addition, initiatives from different concerned departments, including strict policies, are required to prevent exposure to such risk factors. Identifying and ensuring safe drinking water and advising people to drink water sufficiently, especially during hot climates, would help avoid CKDu significantly. More attention could be focused, especially on the regions that are hotspots for CKDu.

## Figures and Tables

**Table 1:** Authors and study information.

Authors	Country	Year	Study setting	LMICs/HMICs	Design	Sample Size	Male	Female	Age
ChannaJayasumana et al. [20]	Sri Lanka	2015	Hospital	LMICs	Case-control	305	98	82	Mean (SD) = 45.51 (19.78) years
VanDervort DR. [21]	El-Salvador	2014	Community	LMICs	Cross-sectional	24,726	NA	NA	NA
SA. Hamilton et al. [22]	Malawi	2020	Community	LMICs	Cross-sectional	821	317	504	Mean (SD) = 33.5 (12.7) years
M Gonzalez et al. [23]	Nicaragua	2018	Community	LMICs	Cohort	350	263	87	Age range = 18–30 years, Mean (SD) = 23.9 (3.7) years
E Siriwardhana et al. [24]	Sri Lanka	2015	Community	LMICs	Case-control	200	59	41	Mean (SD) = 47.8 (9.6) years
N Jayatilake et al. [16]	Sri Lanka	2013	Community	LMICs	cross-sectional	627	Not mentioned	Not mentioned	Mean (SD) = 39.1 (14.2) years
S Nanayakkara [25]	Sri Lanka	2014	Community	LMICs	Case-control	311	311 (total male)	0	Mean (SD) =46.6 (9.0) years
L Lopez et al. [26]	El-Salvador	2014	Hospital	LMICs	Cross-sectional	46	36	10	Mean =45 years
T Rango et al. [27]	Sri Lanka	2015	Community	LMICs	Case-control	134	82	52	Mean (SD)= 37.5 (16.6) years
K Gobalarajah et al. [28]	Sri Lanka	2020	Community	LMICs	Cross-sectional	35	28	7	Age range = 30–80 years
S Mascarenhas et al. [29]	India	2017	Community	LMICs	Cohort	266	58	56	Not mentioned
JM jayaseka ra et al. [30]	Sri Lanka	2013	Community	LMICs	Cross-sectional	863	609	254	Mean (SD)= 54.7 (8) years
E Siriwardhana et al. [31]	Sri Lanka	2014	Hospital	LMICs	case-control	200	59	41	Mean (SD)= 46.3 ( 5.9) years
MA Jayasumana [32]	Sri Lanka	2013	Community	LMICs	Case-control	305	Not mentioned	Not mentioned	Not mentioned
R Osorio et al. [33]	Mexico	2012	Community-based	LMICs	cross-sectional	90	20	70	Mean (SD) = 40.9 (12.9) years
X Xing et al. [34]	China	2015	Hospital	LMICs	Cross-sectional	700	150	91	Mean (SD) = 45.02 (15.97) years
R Chandrajith et al. [35]	Sri Lanka	2010	Community	LMICs	case-control	135	NA	NA	NA
M Selvarajah et al. [36]	Sri Lanka	2016	Hospital	LMICs	Cross-sectional	125	92	33	Mean (SD) = 46.2 (11.64)years
Nanayakkara et al. [37]	Sri Lanka	2012	Community	LMICs	Case control	237	73	33	Mean (SD) = [stage1 = 29 (12), stage2 = 45 (7), stage3 = 44 (13), stage4 = 50 (7), stage5 =4 9 (14)]
N Athuraliya et al [38]	Sri Lanka	2011	Community	LMICs	Cross-sectional	6153	66	43	Mean (SD) = 45.05 (14.79) years
de Silva MW et al. [39]	Sri Lanka	2017	Community	LMICs	Case-control	548	184	90	Mean (SD) = 56.1 (10.9) years
S Wijetunge et al. [40]	Sri Lanka	2013	Community	LMICs	Retrospective cohort	211	153	58	Mean (SD) = 36.8 (14) years
Siddarth et al. [41]	India	2013	Hospital	LMICs	case-control	668	170	164	Mean (SD) = 46.0 (7.3) years
B Guttierrez et al. [42]	Mexico	2013	Hospital	LMICs	Case-control	235	178	57	Mean (SD) = 29.24 (15.48) years
S Sayanthooran et al. [43]	Sri Lanka	2017	Community	LMICs	Case-control	60	51	9	Mean age = 51 (12) years

**Table 2:** Study information and co-morbidity status of included participants.

Authors	Excluded Comorbidity to Define CKDu	Comorbidity Status
ChannaJayasumana et al. [20]	DM or chronic and/or severe HTN, history of snakebite, urological disease of known etiology, glomerulonephritis	Not mentioned
VanDervort DR. [21]	Not mentioned	Not mentioned
SA. Hamilton et al. [22]	DM, HTN, and Heavy Proteinuria	Obese = 54, HIV positive = 3, Overweight = 177
M Gonzalez et al. [23]	Self-reported CKD, DM, or HTN	Not mentioned
E Siriwardhana et al. [24]	DM, long-standing HTN, glomerular nephritis, urolithiasis, congenital kidney diseases, history of snake bite and leptospirosis	Not mentioned
N Jayatilake et al. [16]	Glomerulonephritis, pyelonephritis, renal calculi or snake bite, hypertension	Not mentioned
S Nanayakkara [25]	History of DM and HbA1c >6.5% or HTNor other known renal diseases such as autoimmune diseases, glomerular nephritis, Fanconi syndrome or IgA nephropathy	NCDs = 43%
L Lopez et al. [26]	HTN, DM, glomerulopathies, polycystic kidney disease and obstructive kidney disease, HIV positivity	Glomerulomegally(73.3%), Tubular atrophy(89.1%), Mono nuclear inflammatory infiltration, Intimal proliferation, thickening of the tunica media in blood vessels
T Rango et al. [27]	Not mentioned	Normal weight =72 , Underweight = 34). Overweight = 27, and Obese= 1.
K Gobalarajah et al. [28]	The patients’ disease history revealed that they were secondarily diagnosed with DM & HTN only after they developed CKDu.	DM & HTN, but these are secondarily developed.
S Mascarenhas et al. [29]	DM & HTN	
JM jayaseka ra et al. [30]	DM, HTN, UTI or other renal diseases in the history	Not mentioned
E Siriwardhana et al. [31]	DM, long-standing HTN, glomerular nephritis, urolithiasis, and congenital kidney diseases and having a history of snake bite and leptospirosis	Not mentioned
MA Jayasumana [32]	Not mentioned	Not mentioned
R Osorio et al. [33]	Antecedents of renal disease, DM, HTN, UTI	Not mentioned
X Xing et al. [34]	Secondary renal damage, chronic glomerulonephritis, nephritic syndrome, polycystic kidney disease	Hepatitis, tuberculosis, acute and chronic glomerulonephritis, respiratory infections, Urinary calculus and urinary tract infection (UTI), hydronephrosis(50 individuals)
R Chandrajith et al. [35]	Not mentioned	Anaemia is mild in the early stage of CKD, HTN in the late stage, edema is a late feature.Tubular atrophy and glomerular sclerosis
M Selvarajah et al. [36]	DM, chronic or severe HTN, snake bite, glomerulonephritis or urological diseases, active renal or peri-renal infection, structural and anatomical renal abnormalities of the kidney, cysts and renal masses and those with a solitary kidney, coagulopathy, and uncontrolled HTN.	Not mentioned
Nanayakkara et al. [37]	Not clearly stated	Not mentioned
N Athuraliya et al. [38]	HTN, DM	Co-morbidity status among CKDu: Not Mentioned
de Silva MW et al. [39]	DM, HTN, UTI or other diseases likely toaffect renal function	Interstitial nephritis in all CKDu cases
S Wijetunge et al. [40]	DM and long-standingessential HTN	Glomerular sclerosis (GS), Interstitial fibrosis (IF), Interstitial inflammation (II), Tubular atrophy (TA) and Hypertension associated changes in blood vessels
Siddarth et al. [41]	DM and any other known causes of CKD such as chronic glomerulonephritis, hypertensive nephrosclerosis, autosomal dominant polycystic kidney disease, chronic tubulointerstitial nephropathy or evidence of systemic/local (UTI) infection	Not mentioned
B Guttierrez et al. [42]	T2DM, essential HTN, glomerulonephritis, infections, drugs	Not mentioned
S Sayanthooran et al. [43]	DM, chronic or severe HTN snake bite, glomerulonephritis or urological diseases	Asthma = 3

T2DM: type 2 diabetes mellitus, CKD: chronic kidney disease, HTN: hypertension, DM: diabetes mellitus, NCDs non-communicable diseases, UTI: urinary tract infection.

**Table 3:** Diagnosis and dialysis status of study participants.

Authors Name	Diagnosis	Cutoff eGFR during the Inclusion of the Study Participants	Dialysis Status	How Is the Diagnosis Confirmed
ChannaJayasumana et al. [20]	CKDu	Less than 90	Not mentioned	Prediagnosed CKD
VanDervort DR. [21]		Not mentioned	Not mentioned	ICD-10 definitions were used to classify CKD and ESRD
SA.Hamilton et al. [22]	CKDu	<90 mL/min/1.73 m^2^	Not mentioned	Heavily proteinuric if the albumin: creatinine ratio (ACR) was ≥30 mg/mmol
M Gonzalez et al. [23]	MeN	>90 mL/min/1.73 m^2^	No	Serum creatinine, cysteine C
E.Siriwardhana et al. [24]	CKDu	<60 mL/min	Not mentioned	Proteinuria, elevated levels of serum creatinine and abdominal ultrasound scan reports
N Jayatilake et al. [16]	CKDu	≤90 mL/min/1.73 m^2^	Not mentioned	Renal biopsy.
S.Nanayakkara [25]	CKDu	Not mentioned	Not mentioned	Not mentioned
L Lopez et al. [26]	CKDu	89 mL/min/ 1.73 m^2^ to 30 mL/min/ 1.73 m^2^	Not mentioned	eGFR calculation.
T Rango et al. [27]	CKDu	Not mentioned	Not mentioned	Data brought from health centers
K Gobalarajah et al. [28]	CKDu	Not mentioned	Not mentioned	Not mentioned
S Mascarenhas et al. [29]	CKDu	Not mentioned	Not mentioned	Serum creatinine, eGFR calculation, Sr urea, Plasma albumin, Sr uric acid, FBS
JM jayaseka ra et al. [30]	CKDu	Not mentioned	Not mentioned	Presence of urinary protein one plus or more in sulphosalicylic acid test on two occasions, and the presence of radiological/pathological evidence of chronic kidney disease.
E Siriwardhana et al. [31]	CKDu	Not mentioned	Not mentioned	Presence of proteinuria, elevated levels of serum Creatinine (>1.2 mg/dL) and confirmed abdominal ultrasound scan /renal biopsy reports
MA Jayasumana [32]	CKDu	Not mentioned	Not mentioned	Not mentioned
R Osorio et al. [33]	CKDu	<60 mL/min	Not mentioned	Not mentioned
X Xing et al. [34]	CKDu	s ≥ 90 mL/min/1.73 m^2^	73%	Kidney damage is more than or equal to 3 years.
R Chandrajith et al. [35]	CKDu	Not mentioned	Not mentioned	Not mentioned
M Selvarajah et al. [36]	CKDu	<90 mL/min/1.73 m^2^	Not mentioned	Renal biopsy and pre-diagnosed, and the presence of proteinuria
Nanayakkara et al. [37]	CKDu	≤90 mL/min/1.73 m^2^	Not mentioned	House-to-house screening using dipstick proteinuria on threeoccasions
N Athuraliya et al. [38]	CKDu	Not mentioned	Not mentioned	Proteinurea
de Silva MW et al [39]	CKDu with interstitial nephritis	Not mentioned	Not mentioned	Who showed proteinuria on two occasions along with other features of kidney disease (decreased eGFR, radiological changes in kidney size, increased cortical echogenicity, and loss of corticomedullary demarcation)
S Wijetunge et al. [40]	CKDu	Not mentioned	Not included	Dipstick albuminuria positive(compulsory), renal biopsy(in patients of whom positive albuminuria +1 or above on at least two consecutive dipstick tests and proteinuria more than 500 mg/24 h, or proteinuria less than 500 mg/24 h with haematuria, or proteinuria less than 500 mg/24 h with renal insufficiency)
Siddarth et al. [41]	CKDu	<90 mL/min/1.73 m^2^	Not mentioned	Not mentioned
B Guttierrez et al. [42]	ESRD of unknown causes	Less than 15 mL/min	Peritoneal dialysis, automated peritoneal dialysis, hemodialysis = 32%	eGFR calculation
S Sayanthooran et al. [43]	CKDu	≤90 mL/min/1.73 m^2^	Not mentioned	EGFR calculation

**Table 4:** Associated factors and types of analysis.

Authors	Analysis for Association	Associated Factors
ChannaJayasumana et al. [20]	Unadjusted odds ratio, Mann–Whitney test	Farming, use of herbicide during farming, Well
VanDervort DR. [21]	Geographically weighted regression	People residing near sugarcane agriculture field areas based on the global information system (GIS) technique
SA. Hamilton et al. [22]	Linear regression, logistic regression	Regular meat eater, Age per 10-year increase, Gender
M Gonzalez et al. [23]	Compared with rapid decline, probability-weighted logistic regression	Current or former employees of banana plantations, sugarcane farming, cane cutters, seeders, duration of farming, Use of Herbicide during farming, substance abuse, availability of shade during working hours, people who frequently work in a hot environment, Consuming pipe water supply
E Siriwardhana et al. [24]	Linear logistic model analysis	Farming, paddy farming, Herbicide, substance abuse, Working under the sun for more than 6 h per day and consuming less than 3 litres of water per day, consumption of NSAID drugs, history of malaria
N Jayatilake et al. [16]	Logistic regression	Farming, Substance abuse, Smoking, Age more than 39 years, Gender
S Nanayakkara [25]	Univariate and multiple logistic analyses, Student-Newman-Keuls (SNK) multiple range test, student t-test	History of Malaria
L Lopez et al. [26]	F test	Sugarcane workers, tobacco use
T Rango et al. [27]	Logistic regression	Presence of trace elements such as cadmium (Cd), arsenic (As), lead (Pb), and uranium (U) in the available water sources
K Gobalarajah et al. [28]	Regression analysis between creatinine of CKDu and explanatory variables	Dissolved solids and Arsenic, Phosphate content
S Mascarenhas et al. [29]	Descriptive statistic was performed (Differences at *p* < 0.05 were considered to be significant.)	Blood lead level in affected individuals, pH of groundwater of endemic areas and its seasonal variation
JM jayaseka ra et al. [30]	GIS distribution mapping	Gender, age group over 40 years, farmers. The source of drinking water (shallow wells, tube wells and water reservoirs), patients who consumed boiled water, Clustering of the disease
E Siriwardhana et al. [31]	Fisher’s exact test, chi-square test v	Urine B2M level, Food habits, Consumption of foods which are locally produced, Surface water used for consumption by the local community
MA Jayasumana [32]	Logistic regression and proportion	Arsenic level, Chronic Arsenic Toxicity
R Osorio et al. [33]	Linear correlation analysis with Pearson and coefficient of determination	Age more than 50 years.
X Xing et al. [34]	Multiple logistic regression.	Age more than 60 years, Nephrotoxic drugs, Alcohol consumption
R Chandrajith et al. [35]	T-test	Heavy metals in water bodies (Cadmium, fluoride level)
M Selvarajah et al. [36]	Chi-square test and descriptive statistics	Age, Gender, Family history of CKD
Nanayakkara et al. [37]	Spearman’s rank correlation, Welch’s t-test	N-acetyl-B-D-glucosaminidase (NAG) and alpha1-microglobulin (A1M) excretion
N Athuraliya et al. [38]	Logistic regression	a1-microglobulin (A1M) excretion
de Silva MW et al. [39]	Correlation	Gender (wage labourers)
S Wijetunge et al. [40]	Corellation test	Consuming water from abandoned water sources and well water, the presence of heavy metals in abandoned wells [such as Calcium (Ca), Magnesium (Mg), Barium (Ba), Strontium (Sr), Iron (Fe), Titanium (Ti), and Vanadium (V)], family history of CKD
Siddarth et al. [41]	Chi square test, multinomial logistic regression	Genetic factor
B Guttierrez et al. [42]	χ2 test or Fisher’s exact test, binary logistic regression	Genetic factors
S Sayanthooran et al. [43]	Logistic regression	Genetic factors

## Data Availability

Data can be accessed through reasonable request to corresponding author.

## Supplementary Materials

The following supporting information can be downloaded at: www.mdpi.com/xxx/s1,Table S1: Search strategy. Table S2: Quality check sheet. Table S3: Excluded articles. Table S4: Prisma checklist.

## Abbreviations

CKDu	Chronic kidney disease of unknown etiology
HTN	Hypertension
DM	Diabetes mellitus
B_2_M	Beta-2 microglobulin
A1M	Alpha1-microglobulin, and NAG:N-acetyl-B-D-glucosaminidase
