# Supplementary material for: Factors Associated with Chronic Kidney Disease of Unknown Etiology (CKDu): A Systematic Review
Source: Healthcare (Basel). 2023 Feb 13;11(4):551. doi: 10.3390/healthcare11040551 (PMC9956943; doi:10.3390/healthcare11040551)
Supplement: Supplementary file 1 [file healthcare-11-00551-s001.zip › Table S1.pdf]

TABLE S1

concept	Mesh term	Key term
etiology	“condition, social” [Mesh]<br><br>“Environmental factors”[Mesh]<br><br>“Demographical factors”[Mesh]<br><br>“Environmental Exposure*”[Mesh]<br><br>“Demographic Factor*”[Mesh]<br><br>“Social Factor*”[Mesh]	“Etiological factors” [tiab]<br><br>Etiology[tiab]<br><br>“Etiological factors” [tiab]<br><br>“Factor*, Social” [tiab]<br><br>“Social Factor*”[tiab]<br><br>“Quality, Water” [tiab]<br><br>“Monitor*, Environmental” [tiab]<br><br>“Environmental Surveillance” [tiab]<br><br>“Surveillance, Environment*”[tiab]<br><br>“Exposure*, Environmental” [tiab]<br><br>“Environmental monitor*”[tiab]<br><br>“Environmental factor*”[tiab]<br><br>“Factor*,Environmental” [tiab]<br><br>“Biomarker*, Environment*”[tiab]<br><br>“Environmental Biomarker*”[tiab]<br><br>Bioindicator*[tiab]<br><br>“Biological Indicator*”[tiab]<br><br>“Indicator*, Biologic*”[tiab]<br><br>“Exposure*, Environment*”[tiab]<br><br>“Factor*, Demograph*”[tiab]<br><br>“Demographic Factor*”[tiab]

		Demographic*[tiab]<br><br>“Demographic Account*”[tiab]<br><br>“Account*, Demograph*”[tiab]<br><br>“Demographic Analysis” [tiab]<br><br>“Analys*, Demograph” [tiab]<br><br>“Demographic and Health Survey*”[tiab]<br><br>“Family constitution*”[tiab]<br><br>"Constitution*, Family” [tiab]<br><br>“Family Reconstitution*”[tiab]<br><br>“Reconstitution*, Family” [tiab]<br><br>“Impact*, Demographic” [tiab]<br><br>“Demographic Impact*”[tiab]<br><br>“Multiregional Analysis*”[tiab]<br><br>“Analysis*, Multiregional” [tiab]<br><br>“Period Analysis*”[tiab]<br><br>“Analys*, Period*”[tiab]<br><br>“Period Analyse*”[tiab]<br><br>“Population Distribution”[tiab]<br><br>“Distribution*, Population”[tiab]<br><br>“Survey*, Demograph*”[tiab]<br><br>“Demographic Survey*”[tiab]<br><br>“Population density” [tiab]<br><br>“Age distribution*”[tiab]<br><br>“Sex distribution*”[tiab]

		“Residence characteristic*”[tiab]<br><br>“Impact*, Demograph*”[tiab]<br><br>“Demographic Impact*”[tiab]<br><br>“Biomarker*, Environment*”[tiab]<br><br>“Environmental Biomarker*”[tiab]<br><br>Bioindicator*[tiab]<br><br>“Biological Indicator*”[tiab]<br><br>“Indicator*, Biological” [tiab]<br><br>“Socio-economical factor*”[tiab]
riskfactor	“Factor*, Risk”[Mesh]	“Factor*, Risk” [tiab]<br><br>“Risk Factor*”[tiab]<br><br>“Health Correlate*”[tiab]<br><br>“Correlate*, health” [tiab]<br><br>“Risk Score*”[tiab]<br><br>“Risk Score” [tiab]<br><br>“Score*, Risk*”[tiab]<br><br>“Population*, at Risk” [tiab]<br><br>“Populations at Risk*”[tiab]<br><br>“Population at risk*”[tiab]<br><br>“Associated factor*”[tiab]<br>“Associate Factor*”<br><br>“Co-factor*”[tiab]<br><br>“Cofactor*”[tiab]<br><br>“Factor*, connect*”[tiab]<br><br>“Connecting factor*”[tiab]<br><br>“Contributing factor*”[tiab]<br><br>“Determining factor*”[tiab]

		“Contextual factor*”[tiab]<br>“Relevant factor*”[tiab]<br>“Interrelated factor*”[tiab]<br>“Factor*,Determine*”[tiab]<br>“Factor*,contribut*”[tiab]<br>“factor*, relevant*”[tiab]<br>“factor*, interrelate*”[tiab]<br>“Factor*, connect*”[tiab]<br>Determinant*[tiab]<br>Cause*[tiab]<br>Aspect*[tiab]<br>Characteristic* [tiab]<br>Consideration*[tiab]<br>Component*[tiab]<br>“Relevant component*”[tiab]<br>“Interrelated character*”[tiab]<br>“Connected character*”[tiab]<br>“connected component*”[tiab]<br>“Relevant character*”[tiab]<br>“Connected Aspect*”[tiab]<br>“Relevant aspect*”[tiab]<br>“Contextual aspect*”[tiab]
CKD		“Chronic Renal Insufficienc*”[tiab]<br>“Renal Insufficienc*, Chronic” [tiab]

		“Kidney Insufficienc*, Chronic” [tiab]<br><br>“Chronic Kidney Insufficienc*”[tiab]<br><br>“Kidney Insufficience*, Chronic” [tiab]<br><br>“Chronic Kidney Disease*”[tiab]<br><br>“Kidney Disease*”, Chronic” [tiab]<br><br>“Chronic Renal Disease*”[tiab]<br><br>“Disease*, Chronic Renal” [tiab]<br><br>“Chronic-insufficienc*, Kidney” [tiab]<br><br>“Chronic insufficienc*, Kidney” [tiab]<br><br>“Chronic Renal Insufficienc*”[tiab]<br><br>“Chronic-insufficienc*,Renal” [tiab]<br><br>“Chronic Kidney Disease*”[tiab]<br><br>“Disease*, Chronic Kidney” [tiab]<br><br>“Renal Disease*, Chronic”[tiab]<br><br>“End-Stage Kidney Disease*”[tiab]<br><br>“Disease*, End-Stage Kidney” [tiab]<br><br>“End Stage Kidney Disease” [tiab]<br><br>“Kidney Disease*, End-Stage” [tiab]<br><br>“Chronic Kidney Failure”[tiab]<br><br>“End-Stage Renal Disease”

		[tiab]<br><br>“Disease*, End-Stage Renal”[tiab]<br><br>“End Stage Renal Disease*”[tiab]<br><br>“Renal Disease*, End-Stage”[tiab]<br><br>“Renal Disease*, End Stage” [tiab]<br><br>“Renal Failure, End-Stage”[tiab]<br><br>“End-Stage Renal Failure”[tiab]<br><br>“Renal Failure, End Stage”[tiab]<br><br>“Renal Failure, Chronic”[tiab]<br><br>“Chronic Renal Failure”[tiab]<br><br>ESRD[tiab]<br><br>“End stage renal insufficienc*”[tiab]<br><br>“End-stage Renal insufficienc*”[tiab]<br><br>“Renal insufficienc*, end stage” [tiab]<br><br>Renal insufficienc*, end-stage” [tiab]<br><br>“End stage kidney insufficienc*”[tiab]<br><br>“End-stage Kidney insufficienc*”[tiab]<br><br>“Kidney insufficienc*, end stage” [tiab]<br><br>“Kidney insufficienc*, end-stage” [tiab]<br><br>“End-Stage Kidney

		Disease"[tiab]<br><br>"Disease, End-Stage Kidney"[tiab]<br><br>"Glomerulonephritides"[tiab]<br><br>"Kidney Scarring"[tiab]<br><br>"Scarring, Kidney"[tiab]<br><br>"Bright Disease"[tiab]<br><br>"Dermopath*, Nephrogenic Fibros*"[tiab]<br><br>"Fibrosing Dermopath*, Nephrogenic"[tiab]<br><br>"Nephrogenic Fibrosing Dermopath*"[tiab]<br><br>"Nephrogenic Systemic Fibros*" [tiab]<br><br>"Fibros*, Nephrogenic Systemic" [tiab]<br><br>"Systemic Fibros*, Nephrogenic" [tiab]<br><br>"Xanthogranulomatous Pyelonephritis" [tiab]<br><br>"Pyelonephritides, Xanthogranulomatous"[tiab]<br><br>"Xanthogranulomatous Pyelonephritides"[tiab]<br><br>Nephritis[tiab]<br><br>Pyelonephritides[tiab]<br><br>"Pyelonephritis, Acute Necrotizing"[tiab]<br><br>"Necrotizing Pyelonephritis" [tiab]

		Nephroscleroses[tiab]<br><br>“Chronic Urologic Disease” [tiab]<br><br>“Chronic Kidney disorders” [tiab]<br><br>“Chronic Renal Disorders” [tiab]<br><br>“Chronic disorder*, Kidney” [tiab]<br><br>“Chronic disorder*, Renal” [tiab]<br><br>“End stage Renal disorder*”[tiab]<br><br>“End-stage Renal Disorder*”[tiab]<br><br>“End stage Kidney Disorder*”[tiab]<br><br>“End-stage Kidney Disorder*”[tiab]<br><br>“Kidney Disorder*, End stage” [tiab]<br><br>“Renal Disorder*, End stage” [tiab]<br><br>“chronic nephropathy”[tiab]<br><br>“Chronic-nephropathy”[tiab]<br><br>“Chronic Glomerulopathy”[tiab]<br><br>“Chronic Uropathy”[tiab]<br><br>“End stage nephropathy”[tiab]<br><br>“End-stage nephropthy” [tiab]<br><br>“End stage Glomerulopathy” [tiab]<br><br>“End-stage Glomerulopathy” [tiab]<br><br>“Chronic nephritic syndrome”

		[tiab]<br><br>“Chronic Kidney Disease Mineral and Bone Disorder”[tiab]<br><br>“CKD-MBD”[tiab]<br><br>“Renal Osteodystroph*”[tiab]<br><br>“Osteodystroph*, Renal”[tiab]<br><br>“Chronic Renal impairment” [tiab]<br><br>“Chronic Kidney Impairment” [tiab]<br><br>“Impairment of Renal function” [tiab]<br><br>“Impairment of kidney function” [tiab]<br><br>“Impairment of Renal function*, Chronic” [tiab]<br><br>“Impairment of kidney function*, Chronic”[tiab]<br><br>“
Unknown origin		**“Unknown origin”[tiab]**<br>**“Uncertain origin”[tiab]**<br>**“Origin, unknown”[tiab]**<br>**“Origin, uncertain” [tiab]**

		**"Undetermined cause*"[tiab]<br>"Unspecified cause*"[tiab]<br>"Unrevealed cause*"[tiab]<br>"Unrevealed factor*"[tiab]<br>"Unspecified factor*"[tiab]<br>"Factor*,unspecif*"[tiab]<br>"Factor*, undetermined*"[tiab]<br>"Origin, undetermined*"[tiab]<br>"Origin, unspecif*"[tiab]<br>"Origin, unreveal*"[tiab]<br>"Cause*, unreveal*"[tiab]<br>"Cause*, unspecif*"[tiab]<br>"Cause*, undetermined*"[tiab]<br>"Undecided factor" [tiab]<br>"Factor*, undecided*"[tiab]<br>"Cause*, undecided*"[tiab]<br>"Unknown etiolog*"[tiab]<br>"Etiolog*,unknown" [tiab]<br>"Uncertain etiolog*"[tiab]<br>"Unrevealed etiolog*"[tiab]<br>"Undecided etiolog*"[tiab]<br>"Undetermined etiolog*"[tiab]<br>"Etiolog*, uncertain*"[tiab]<br>"Etiolog*, unreveal" [tiab]<br>"Etiolog*, undecide*"[tiab]<br>"Etiolog*, undetermined*"[tiab]<br>" Unknown Associated factor*"[tiab]<br>"Undecided Co-factor*"[tiab]<br>Unknown Cofactor*[tiab]<br>"Unknown Factor*, connect*"[tiab]<br>"Unknown Connecting factor*"[tiab]<br>"Undecided Contributing factor*"[tiab]<br>"Undecided Determining factor*"[tiab]<br>"Undecided Contextual factor*"[tiab]<br>"Undecided Relevant factor*"[tiab]**

<u>MEDLINE database By using Pubmed</u>

(((“Chronic Renal Insufficienc*”[tiab]) OR “Renal Insufficienc*, Chronic” [tiab]) OR “Kidney Insufficienc*, Chronic” [tiab]) OR “Chronic Kidney Insufficienc*”[tiab]) OR “Kidney Insufficience*, Chronic” [tiab]) OR “Chronic Kidney Disease*”[tiab]) OR “Kidney Disease*”, Chronic” [tiab]) OR “Chronic Renal Disease*”[tiab]) OR “Disease*, Chronic Renal” [tiab]) OR “Chronic-insufficienc*, Kidney” [tiab]) OR “Chronic insufficienc*, Kidney” [tiab]) OR “Chronic Renal Insufficienc*”[tiab]) OR “Chronic Kidney Disease*”[tiab]) OR “Disease*, Chronic Kidney” [tiab]) OR “Renal Disease*, Chronic”[tiab]) OR “End-Stage Kidney Disease*”[tiab]) OR “Disease*, End-Stage Kidney” [tiab]) OR “End Stage Kidney Disease” [tiab]) OR “Kidney Disease*, End-Stage” [tiab]) OR “Chronic Kidney Failure”[tiab]) OR “End-Stage Renal Disease” [tiab]) OR “Disease*, End-Stage Renal”[tiab]) OR “End Stage Renal Disease*”[tiab]) OR “Renal Disease*, End-Stage”[tiab]) OR “Renal Disease*, End Stage” [tiab]) OR “Renal Failure, End-Stage”[tiab]) OR “End-Stage Renal Failure”[tiab]) OR “Renal Failure, End Stage”[tiab]) OR “Renal Failure, Chronic”[tiab]) OR “Chronic Renal Failure”[tiab]) OR ESRD[tiab]) OR “End stage renal insufficienc*”[tiab]) OR “End-stage Renal insufficienc*”[tiab]) OR “Renal insufficienc*, end stage” [tiab]) OR Renal insufficienc*, end-stage” [tiab]) OR “End stage kidney insufficienc*”[tiab]) OR “End-stage Kidney insufficienc*”[tiab]) OR “Kidney insufficienc*, end stage” [tiab]) OR “Kidney insufficienc*, end-stage” [tiab]) OR “End-Stage Kidney Disease”[tiab]) OR “Disease, End-Stage Kidney”[tiab]) OR “Glomerulonephritides”[tiab]) OR “Kidney Scarring”[tiab]) OR “Scarring, Kidney”[tiab]) OR “Bright Disease”[tiab]) OR “Dermopath*, Nephrogenic Fibros*”[tiab]) OR “Fibros*, Nephrogenic Systemic” [tiab]) OR “Fibrosing Dermopath*, Nephrogenic”[tiab]) OR “Nephrogenic Systemic Fibros*” [tiab]) OR “Nephrogenic Fibrosing Dermopath*”[tiab]) OR “Xanthogranulomatous Pyelonephritis” [tiab]) OR “Pyelonephritides, Xanthogranulomatous”[tiab]) OR “Xanthogranulomatous Pyelonephritides”[tiab]) OR Nephritis[tiab]) OR Pyelonephritides[tiab]) OR “Pyelonephritis, Acute Necrotizing”[tiab]) OR “Necrotizing Pyelonephritis” [tiab]) OR Nephroscleroses[tiab]) OR “Chronic Urologic Disease” [tiab]) OR “Chronic Kidney disorders” [tiab]) OR “Chronic Renal Disorders” [tiab]) OR “Chronic disorder*, Kidney” [tiab]) OR “Chronic disorder*, Renal” [tiab]) OR “End stage Renal disorder*”[tiab]) OR “End-stage Renal Disorder*”[tiab]) OR “End stage Kidney Disorder*”[tiab]) OR “End-stage Kidney Disorder*”[tiab]) OR “Kidney Disorder*, End stage”[tiab]) OR “Renal Disorder*, End stage” [tiab]) OR “chronic nephropathy”[tiab]) OR “Chronic-nephropathy”[tiab]) OR “Chronic Glomerulopathy”[tiab]) OR “Chronic Uropathy”[tiab]) OR “End stage nephropathy”[tiab]) OR “End-stage nephropthy” [tiab]) OR “End stage Glomerulopathy” [tiab]) OR “End-stage Glomerulopathy” [tiab]) OR “Chronic nephritic syndrome” [tiab]) OR (“Chronic Kidney Disease Mineral and Bone Disorder”[tiab])) OR “CKD-MBD”[tiab]) OR “Renal Osteodystroph*”[tiab]) OR “Osteodystroph*, Renal”[tiab]) OR “Chronic Renal impairment” [tiab]) OR “Chronic Kidney Impairment” [tiab]) OR

“Impairment of Renal function” [tiab]) OR “Impairment of kidney function” [tiab]) OR “ Kidney Osteodystroph*”[tiab]) OR “Osteodystroph*, kIdney”[tiab]) OR “Impairment of Renal function*, Chronic” [tiab]) OR “Impairment of kidney function*, Chronic”[tiab]

UNKNOWN ORIGIN:-

(((((((((((((((((((((((((((((((((((((“Unknown origin”[tiab]) OR “Uncertain origin”[tiab]) OR “Origin, unknown”[tiab]) OR “Origin, uncertain” [tiab]) OR “Undetermined cause*”[tiab]) OR “Unspecified cause*”[tiab]) OR “Unrevealed cause*”[tiab]) OR “Unrevealed factor*”[tiab]) OR “Unspecified factor*”[tiab]) OR “Factor*,unspecif*”[tiab]) OR “Factor*, undetermined*”[tiab]) OR “Origin, undetermined*”[tiab]) OR “Origin, unspecif*”[tiab]) OR “Origin, unreveal*”[tiab]) OR “Cause*, unreveal*”[tiab]) OR “Cause*, unspecif*”[tiab]) OR “Cause*, undetermined*”[tiab]) OR “Undecided factor” [tiab]) OR “Factor*, undecided*”[tiab]) OR “Cause*, undecided*”[tiab]) OR “Unknown etiolog*”[tiab]) OR “Etiolog*,unknown” [tiab]) OR “Uncertain etiolog*”[tiab]) OR “Unrevealed etiolog*”[tiab]) OR “Undecided etiolog*”[tiab]) OR “Undetermined etiolog*”[tiab]) OR “Etiolog*, uncertain*”[tiab]) OR “Etiolog*, unreveal” [tiab]) OR “Etiolog*, undecide*”[tiab]) OR “Etiolog*, undetermined*”[tiab]) OR “ Unknown Associated factor*”[tiab]) OR “Undecided Co-factor*”[tiab]) OR Unknown Cofactor*[tiab]) OR “Unknown Factor*, connect*”[tiab]) OR “Unknown Connecting factor*”[tiab]) OR “Undecided Contributing factor*”[tiab]) OR “Undecided Determining factor*”[tiab]) OR “Undecided Contextual factor*”[tiab]) OR “Undecided Relevant factor*”[tiab]

RISK FACTOR OR ASSOCIATED FACTOR

(((“Factor*, Risk”[Mesh]) OR “Factor*, Risk” [tiab]) OR “Risk Factor*”[tiab]) OR “Health Correlate*”[tiab]) OR “Correlate*, health” [tiab]) OR “Risk Score*”[tiab]) OR “Risk Score” [tiab]) OR “Score*, Risk*”[tiab]) OR “Population*, at Risk” [tiab]) OR “Populations at Risk*”[tiab]) OR “Population at risk*”[tiab]) OR “Associated factor*”[tiab]) OR “Associate Factor*”) OR “Co-factor*”[tiab]) OR “Cofactor*”[tiab]) OR “Factor*, connect*”[tiab]) OR “Connecting factor*”[tiab]) OR “Contributing factor*”[tiab]) OR “Determining factor*”[tiab]) OR “Contextual factor*”[tiab]) OR “Relevant factor*”[tiab]) OR “Interrelated factor*”[tiab]) OR “Factor*,Determine*”[tiab]) OR “Factor*,contribut*”[tiab]) OR “factor*, relevant*”[tiab]) OR “factor*, interrelate*”[tiab]) OR “Factor*, connect*”[tiab]) OR Determinant*[tiab]) OR Cause*[tiab]) OR Aspect*[tiab]) OR Characteristic*[tiab]) OR

Consideration*[tiab]) OR Component*[tiab]) OR “Relevant component*”[tiab]) OR “Interrelated character*”[tiab]) OR “Connected character*”[tiab]) OR “connected component*”[tiab]) OR “Relevant character*”[tiab]) OR “Connected Aspect*”[tiab]) OR “Relevant aspect*”[tiab]) OR “Contextual aspect*”[tiab])) OR ((“condition, social” [Mesh]) OR “Environmental factors”[Mesh]) OR “Demographical factors”[Mesh]) OR “Environmental Exposure*”[Mesh]) OR “Demographic Factor*”[Mesh]) OR “Social Factor*”[Mesh]) OR “Etiological factors” [tiab]) OR Etiology[tiab]) OR “Etiological factors” [tiab]) OR “Factor*, Social” [tiab]) OR “Social Factor*”[tiab]) OR “Quality, Water” [tiab]) OR “Monitor*, Environmental” [tiab]) OR “Environmental Surveillance” [tiab]) OR “Surveillance, Environment*”[tiab]) OR “Exposure*, Environmental” [tiab]) OR “Environmental monitor*”[tiab]) OR “Environmental factor*”[tiab]) OR “Factor*,Environmental” [tiab]) OR “Biomarker*, Environment*”[tiab]) OR “Environmental Biomarker*”[tiab]) OR Bioindicator*[tiab]) OR “Biological Indicator*”[tiab]) OR “Indicator*, Biologic*”[tiab]) OR “Exposure*, Environment*”[tiab]) OR “Factor*, Demograph*”[tiab]) OR “Demographic Factor*”[tiab]) OR Demographic*[tiab]) OR “Demographic Account*”[tiab]) OR “Account*, Demograph*”[tiab]) OR “Demographic Analys*” [tiab]) OR “Analys*, Demograph” [tiab]) OR (“Demographic and Health Survey*”[tiab])) OR “Family constitution*”[tiab]) OR "Constitution*, Family” [tiab]) OR “Family Reconstitution*”[tiab]) OR “Reconstitution*, Family” [tiab]) OR “Impact*, Demographic” [tiab]) OR “Demographic Impact*”[tiab]) OR “Multiregional Analysis*”[tiab]) OR “Analysis*, Multiregional” [tiab]) OR “Period Analysis*”[tiab]) OR “Analys*, Period*”[tiab]) OR “Period Analyse*”[tiab]) OR “Population Distribution”[tiab]) OR “Distribution*, Population”[tiab]) OR “Survey*, Demograph*”[tiab]) OR “Demographic Survey*”[tiab]) OR “Population density” [tiab]) OR “Age distribution*”[tiab]) OR “Sex distribution*”[tiab]) OR “Residence characteristic*”[tiab]) OR “Impact*, Demograph*”[tiab]) OR “Demographic Impact*”[tiab]) OR “Biomarker*, Environment*”[tiab]) OR “Environmental Biomarker*”[tiab]) OR Bioindicator*[tiab]) OR “Biological Indicator*”[tiab]) OR “Indicator*, Biological” [tiab]) OR “Socio-economical factor*”[tiab])

CKD and UNKNOWN ORIGIN RISK FACTOR OR ASSOCIATED FACTOR

((“Chronic Renal Insufficienc*”[tiab]) OR “Renal Insufficienc*, Chronic” [tiab]) OR “Kidney Insufficienc*, Chronic” [tiab]) OR “Chronic Kidney Insufficienc*”[tiab]) OR “Kidney Insufficience*, Chronic” [tiab]) OR “Chronic Kidney Disease*”[tiab]) OR “Kidney Disease*”, Chronic” [tiab]) OR “Chronic Renal Disease*”[tiab]) OR “Disease*, Chronic Renal” [tiab]) OR “Chronic-insufficienc*, Kidney” [tiab]) OR “Chronic insufficienc*, Kidney” [tiab]) OR “Chronic Renal Insufficienc*”[tiab]) OR “Chronic Kidney Disease*”[tiab]) OR “Disease*, Chronic Kidney” [tiab]) OR “Renal Disease*, Chronic”[tiab]) OR “End-Stage Kidney Disease*”[tiab]) OR “Disease*, End-Stage Kidney” [tiab]) OR “End Stage Kidney Disease” [tiab]) OR “Kidney Disease*, End-Stage” [tiab]) OR “Chronic Kidney Failure”[tiab]) OR “End-Stage Renal Disease”

[tiab]) OR “Disease*, End-Stage Renal”[tiab]) OR “End Stage Renal Disease*”[tiab]) OR “Renal Disease*, End-Stage”[tiab]) OR “Renal Disease*, End Stage” [tiab]) OR “Renal Failure, End-Stage”[tiab]) OR “End-Stage Renal Failure”[tiab]) OR “Renal Failure, End Stage”[tiab]) OR “Renal Failure, Chronic”[tiab]) OR “Chronic Renal Failure”[tiab]) OR ESRD[tiab]) OR “End stage renal insufficienc*”[tiab]) OR “End-stage Renal insufficienc*”[tiab]) OR “Renal insufficienc*, end stage” [tiab]) OR Renal insufficienc*, end-stage” [tiab]) OR “End stage kidney insufficienc*”[tiab]) OR “End-stage Kidney insufficienc*”[tiab]) OR “Kidney insufficienc*, end stage” [tiab]) OR “Kidney insufficienc*, end-stage” [tiab]) OR “End-Stage Kidney Disease”[tiab]) OR “Disease, End-Stage Kidney”[tiab]) OR “Glomerulonephritides”[tiab]) OR “Kidney Scarring”[tiab]) OR “Scarring, Kidney”[tiab]) OR “Bright Disease”[tiab]) OR “Dermopath*, Nephrogenic Fibros*”[tiab]) OR “Fibros*, Nephrogenic Systemic” [tiab]) OR “Fibrosing Dermopath*, Nephrogenic”[tiab]) OR “Nephrogenic Systemic Fibros*” [tiab]) OR “Nephrogenic Fibrosing Dermopath*”[tiab]) OR “Xanthogranulomatous Pyelonephritis” [tiab]) OR “Pyelonephritides, Xanthogranulomatous”[tiab]) OR “Xanthogranulomatous Pyelonephritides”[tiab]) OR Nephritis[tiab]) OR Pyelonephritides[tiab]) OR “Pyelonephritis, Acute Necrotizing”[tiab]) OR “Necrotizing Pyelonephritis” [tiab]) OR Nephroscleroses[tiab]) OR “Chronic Urologic Disease” [tiab]) OR “Chronic Kidney disorders” [tiab]) OR “Chronic Renal Disorders” [tiab]) OR “Chronic disorder*, Kidney” [tiab]) OR “Chronic disorder*, Renal” [tiab]) OR “End stage Renal disorder*”[tiab]) OR “End-stage Renal Disorder*”[tiab]) OR “End stage Kidney Disorder*”[tiab]) OR “End-stage Kidney Disorder*”[tiab]) OR “Kidney Disorder*, End stage”[tiab]) OR “Renal Disorder*, End stage” [tiab]) OR “chronic nephropathy”[tiab]) OR “Chronic-nephropathy”[tiab]) OR “Chronic Glomerulopathy”[tiab]) OR “Chronic Uropathy”[tiab]) OR “End stage nephropathy”[tiab]) OR “End-stage nephropthy” [tiab]) OR “End stage Glomerulopathy” [tiab]) OR “End-stage Glomerulopathy” [tiab]) OR “Chronic nephritic syndrome” [tiab]) OR (“Chronic Kidney Disease Mineral and Bone Disorder”[tiab])) OR “CKD-MBD”[tiab]) OR “Renal Osteodystroph*”[tiab]) OR “Osteodystroph*, Renal”[tiab]) OR “Chronic Renal impairment” [tiab]) OR “Chronic Kidney Impairment” [tiab]) OR “Impairment of Renal function” [tiab]) OR “Impairment of kidney function” [tiab]) OR “ Kidney Osteodystroph*”[tiab]) OR “Osteodystroph*, kIdney”[tiab]) OR “Impairment of Renal function*, Chronic” [tiab]) OR “Impairment of kidney function*, Chronic”[tiab])) AND ((((((((((((((((((((((((((((((((((((((“Unknown origin”[tiab]) OR “Uncertain origin”[tiab]) OR “Origin, unknown”[tiab]) OR “Origin, uncertain” [tiab]) OR “Undetermined cause*”[tiab]) OR “Unspecified cause*”[tiab]) OR “Unrevealed cause*”[tiab]) OR “Unrevealed factor*”[tiab]) OR “Unspecified factor*”[tiab]) OR “Factor*,unspecif*”[tiab]) OR “Factor*, undetermined*”[tiab]) OR “Origin, undetermined*”[tiab]) OR “Origin, unspecif*”[tiab]) OR “Origin, unreveal*”[tiab]) OR “Cause*, unreveal*”[tiab]) OR “Cause*, unspecif*”[tiab]) OR “Cause*, undetermined*”[tiab]) OR “Undecided factor” [tiab]) OR “Factor*, undecided*”[tiab]) OR “Cause*, undecided*”[tiab]) OR “Unknown etiolog*”[tiab]) OR “Etiolog*,unknown” [tiab]) OR “Uncertain etiolog*”[tiab]) OR “Unrevealed etiolog*”[tiab]) OR “Undecided etiolog*”[tiab]) OR “Undetermined etiolog*”[tiab]) OR “Etiolog*, uncertain*”[tiab]) OR “Etiolog*, unreveal” [tiab]) OR “Etiolog*, undecide*”[tiab]) OR “Etiolog*, undetermined*”[tiab]) OR “ Unknown Associated factor*”[tiab]) OR “Undecided Co-factor*”[tiab]) OR Unknown Cofactor*[tiab]) OR “Unknown Factor*, connect*”[tiab]) OR

“Unknown Connecting factor*”[tiab]) OR “Undecided Contributing factor*”[tiab]) OR “Undecided Determining factor*”[tiab]) OR “Undecided Contextual factor*”[tiab]) OR “Undecided Relevant factor*”[tiab])) AND (((“Factor*, Risk”[Mesh]) OR “Factor*, Risk” [tiab]) OR “Risk Factor*”[tiab]) OR “Health Correlate*”[tiab]) OR “Correlate*, health” [tiab]) OR “Risk Score*”[tiab]) OR “Risk Score” [tiab]) OR “Score*, Risk*”[tiab]) OR “Population*, at Risk” [tiab]) OR “Populations at Risk*”[tiab]) OR “Population at risk*”[tiab]) OR “Associated factor*”[tiab]) OR “Associate Factor*”) OR “Co-factor*”[tiab]) OR “Cofactor*”[tiab]) OR “Factor*, connect*”[tiab]) OR “Connecting factor*”[tiab]) OR “Contributing factor*”[tiab]) OR “Determining factor*”[tiab]) OR “Contextual factor*”[tiab]) OR “Relevant factor*”[tiab]) OR “Interrelated factor*”[tiab]) OR “Factor*,Determine*”[tiab]) OR “Factor*,contribut*”[tiab]) OR “factor*, relevant*”[tiab]) OR “factor*, interrelate*”[tiab]) OR “Factor*, connect*”[tiab]) OR Determinant*[tiab]) OR Cause*[tiab]) OR Aspect*[tiab]) OR Characteristic*[tiab]) OR Consideration*[tiab]) OR Component*[tiab]) OR “Relevant component*”[tiab]) OR “Interrelated character*”[tiab]) OR “Connected character*”[tiab]) OR “connected component*”[tiab]) OR “Relevant character*”[tiab]) OR “Connected Aspect*”[tiab]) OR “Relevant aspect*”[tiab]) OR “Contextual aspect*”[tiab])) OR ((“condition, social” [Mesh]) OR “Environmental factors”[Mesh]) OR “Demographical factors”[Mesh]) OR “Environmental Exposure*”[Mesh]) OR “Demographic Factor*”[Mesh]) OR “Social Factor*”[Mesh]) OR “Etiological factors” [tiab]) OR Etiology[tiab]) OR “Etiological factors” [tiab]) OR “Factor*, Social” [tiab]) OR “Social Factor*”[tiab]) OR “Quality, Water” [tiab]) OR “Monitor*, Environmental” [tiab]) OR “Environmental Surveillance” [tiab]) OR “Surveillance, Environment*”[tiab]) OR “Exposure*, Environmental” [tiab]) OR “Environmental monitor*”[tiab]) OR “Environmental factor*”[tiab]) OR “Factor*,Environmental” [tiab]) OR “Biomarker*, Environment*”[tiab]) OR “Environmental Biomarker*”[tiab]) OR Bioindicator*[tiab]) OR “Biological Indicator*”[tiab]) OR “Indicator*, Biologic*”[tiab]) OR “Exposure*, Environment*”[tiab]) OR “Factor*, Demograph*”[tiab]) OR “Demographic Factor*”[tiab]) OR Demographic*[tiab]) OR “Demographic Account*”[tiab]) OR “Account*, Demograph*”[tiab]) OR “Demographic Analys*” [tiab]) OR “Analys*, Demograph” [tiab]) OR (“Demographic and Health Survey*”[tiab])) OR “Family constitution*”[tiab]) OR "Constitution*, Family” [tiab]) OR “Family Reconstitution*”[tiab]) OR “Reconstitution*, Family” [tiab]) OR “Impact*, Demographic” [tiab]) OR “Demographic Impact*”[tiab]) OR “Multiregional Analysis*”[tiab]) OR “Analysis*, Multiregional” [tiab]) OR “Period Analysis*”[tiab]) OR “Analys*, Period*”[tiab]) OR “Period Analyse*”[tiab]) OR “Population Distribution”[tiab]) OR “Distribution*, Population”[tiab]) OR “Survey*, Demograph*”[tiab]) OR “Demographic Survey*”[tiab]) OR “Population density” [tiab]) OR “Age distribution*”[tiab]) OR “Sex distribution*”[tiab]) OR “Residence characteristic*”[tiab]) OR “Impact*, Demograph*”[tiab]) OR “Demographic Impact*”[tiab]) OR “Biomarker*, Environment*”[tiab]) OR “Environmental Biomarker*”[tiab]) OR Bioindicator*[tiab]) OR “Biological Indicator*”[tiab]) OR “Indicator*, Biological” [tiab]) OR “Socio-economical factor*”[tiab]))

TI “Chronic Renal Insufficienc*” OR TI “Renal Insufficienc*, Chronic” OR TI “Kidney Insufficienc*, Chronic” OR TI “Chronic Kidney Insufficienc*” OR TI “Kidney Insufficience*, Chronic” OR TI “Chronic Kidney Disease*” OR TI “Kidney Disease*”, Chronic” OR TI “Chronic Renal Disease*” OR TI “Disease*, Chronic Renal” OR TI “Chronic-insufficienc*, Kidney” OR TI “Chronic insufficienc*, Kidney” OR TI “Chronic Renal Insufficienc*” OR TI “Chronic Kidney Disease*” OR TI “Disease*, Chronic Kidney” OR TI “Renal Disease*, Chronic” OR TI “End-Stage Kidney Disease*” OR TI “Disease*, End-Stage Kidney” OR TI “End Stage Kidney Disease” OR TI “Kidney Disease*, End-Stage” OR TI “Chronic Kidney Failure” OR TI “End-Stage Renal Disease” OR TI “Disease*, End-Stage Renal” OR TI “End Stage Renal Disease*” OR TI “Renal Disease*, End-Stage” OR TI “Renal Disease*, End Stage” OR TI “Renal Failure, End-Stage” OR TI “End-Stage Renal Failure” OR TI “Renal Failure, End Stage” OR TI “Renal Failure, Chronic” OR TI “Chronic Renal Failure” OR AB “Chronic Renal Insufficienc*” OR AB “Renal Insufficienc*, Chronic” OR AB “Kidney Insufficienc*, Chronic” OR AB “Chronic Kidney Insufficienc*” OR AB “Kidney Insufficience*, Chronic” OR AB “Chronic Kidney Disease*” OR AB “Kidney Disease*”, Chronic” OR AB “Chronic Renal Disease*” OR AB “Disease*, Chronic Renal” OR AB “Chronic-insufficienc*, Kidney” OR AB “Chronic insufficienc*, Kidney” OR AB “Chronic Renal Insufficienc*” OR AB “Chronic Kidney Disease*” OR AB “Disease*, Chronic Kidney” OR AB “Renal Disease*, Chronic” OR AB “End-Stage Kidney Disease*” OR AB “Disease*, End-Stage Kidney” OR AB “End Stage Kidney Disease” OR AB “Kidney Disease*, End-Stage” OR AB “Chronic Kidney Failure” OR AB “End-Stage Renal Disease” OR AB “Disease*, End-Stage Renal” OR AB “End Stage Renal Disease*” OR AB “Renal Disease*, End-Stage” OR AB “Renal Disease*, End Stage” OR AB “Renal Failure, End-Stage” OR AB “End-Stage Renal Failure” OR AB “Renal Failure, End Stage” OR AB “Renal Failure, Chronic” OR AB “Chronic Renal Failure”

CINHAL database using EBSCOHOST

TI “Chronic Renal Insufficienc*” OR TI “Renal Insufficienc*, Chronic” OR TI “Kidney Insufficienc*, Chronic” OR TI “Chronic Kidney Insufficienc*” OR TI “Kidney Insufficience*, Chronic” OR TI “Chronic Kidney Disease*” OR TI “Kidney Disease*”, Chronic” OR TI “Chronic Renal Disease*” OR TI “Disease*, Chronic Renal” OR TI “Chronic-insufficienc*, Kidney” OR TI “Chronic insufficienc*, Kidney” OR TI “Chronic Renal Insufficienc*” OR TI “Chronic Kidney Disease*” OR TI “Disease*, Chronic Kidney” OR TI “Renal Disease*, Chronic” OR TI “End-Stage Kidney Disease*” OR TI “Disease*, End-Stage Kidney” OR TI “End Stage Kidney Disease” OR TI “Kidney Disease*, End-Stage” OR TI “Chronic Kidney Failure” OR TI “End-Stage Renal Disease” OR TI “Disease*, End-Stage Renal” OR TI “End Stage Renal Disease*” OR TI “Renal Disease*, End-Stage” OR TI “Renal Disease*, End Stage” OR TI “Renal Failure, End-Stage” OR TI “End-Stage Renal Failure” OR TI “Renal Failure, End Stage” OR TI “Renal Failure, Chronic” OR TI “Chronic Renal Failure” OR AB “Chronic Renal Insufficienc*” OR AB “Renal Insufficienc*, Chronic” OR AB “Kidney Insufficienc*, Chronic” OR AB “Chronic Kidney Insufficienc*” OR AB “Kidney Insufficience*, Chronic” OR AB “Chronic Kidney Disease*” OR AB “Kidney Disease*”, Chronic” OR AB “Chronic Renal Disease*” OR AB “Disease*, Chronic Renal” OR AB “Chronic-insufficienc*, Kidney” OR AB “Chronic insufficienc*, Kidney” OR AB “Chronic Renal Insufficienc*” OR AB “Chronic Kidney Disease*” OR AB “Disease*, Chronic Kidney” OR AB “Renal Disease*, Chronic” OR AB “End-Stage Kidney Disease*” OR AB “Disease*, End-Stage Kidney” OR AB “End Stage Kidney Disease” OR AB “Kidney Disease*, End-Stage” OR AB “Chronic Kidney Failure” OR AB “End-Stage Renal Disease” OR AB “Disease*, End-Stage Renal” OR AB

“End Stage Renal Disease*” OR AB “Renal Disease*, End-Stage” OR AB “Renal Disease*, End Stage” OR AB “Renal Failure, End-Stage” OR AB “End-Stage Renal Failure” OR AB “Renal Failure, End Stage” OR AB “Renal Failure, Chronic” OR AB “Chronic Renal Failure”

AB “Unknown origin” OR AB “Uncertain origin” OR AB “Origin, unknown” OR AB “Origin, uncertain” OR AB “Undetermined cause*” OR AB “Unspecified cause*” OR AB “Unrevealed cause*” OR AB “Unrevealed factor*” OR AB “Unspecified factor*” OR AB “Factor*,unspecif*” OR AB “Factor*, undetermined*” OR AB “Origin, undetermined*” OR AB “Origin, unspecif*” OR AB “Origin, unreveal*” OR AB “Cause*, unreveal*” OR AB “Cause*, unspecif*” OR AB “Cause*, undetermined*” OR AB “Undecided factor” OR AB “Factor*, undecided*” OR AB “Cause*, undecided*” OR AB “Unknown etiolog*” OR AB “Etiolog*,unknown” OR AB “Uncertain etiolog*” OR AB “Unrevealed etiolog*” OR AB “Undecided etiolog*” OR AB “Undetermined etiolog*” OR AB “Etiolog*, uncertain*” OR AB “Etiolog*, unreveal” OR AB “Etiolog*, undecide*” OR AB “Etiolog*, undetermined*” OR AB “Unknown Associated factor*” OR AB “Undecided Co-factor*” OR AB Unknown Cofactor* OR AB “Unknown Factor*, connect*” OR AB “Unknown Connecting factor*” OR AB “Undecided Contributing factor*” OR AB “Undecided Determining factor*” OR AB “Undecided Contextual factor*” OR AB “Undecided Relevant factor*” OR TI “Unknown origin” OR TI “Uncertain origin” OR TI “Origin, unknown” OR TI “Origin, uncertain” OR TI “Undetermined cause*” OR TI “Unspecified cause*” OR TI “Unrevealed cause*” OR TI “Unrevealed factor*” OR TI “Unspecified factor*” OR TI “Factor*,unspecif*” OR TI “Factor*, undetermined*” OR TI “Origin, undetermined*” OR TI “Origin, unspecif*” OR TI “Origin, unreveal*” OR TI “Cause*, unreveal*” OR TI “Cause*, unspecif*” OR TI “Cause*, undetermined*” OR TI “Undecided factor” OR TI “Factor*, undecided*” OR TI “Cause*, undecided*” OR TI “Unknown etiolog*” OR TI “Etiolog*,unknown” OR TI “Uncertain etiolog*” OR TI “Unrevealed etiolog*” OR TI “Undecided etiolog*” OR TI “Undetermined etiolog*” OR TI “Etiolog*, uncertain*” OR TI “Etiolog*, unreveal” OR TI “Etiolog*, undecide*” OR TI “Etiolog*, undetermined*” OR TI “Unknown Associated factor*” OR TI “Undecided Co-factor*” OR TI “Unknown Cofactor* OR TI “Unknown Factor*, connect*” OR TI “Unknown Connecting factor*” OR TI “Undecided Contributing factor*” OR TI “Undecided Determining factor*” OR TI “Undecided Contextual factor*” OR TI “Undecided Relevant factor*”

OVID:

((Chronic Renal Insufficienc) OR (Renal Insufficienc, Chronic) OR (Kidney Insufficienc Chronic) OR (Chronic Kidney Insufficienc) OR (Kidney Insufficience Chronic) OR (Chronic Kidney Disease) OR (Kidney Disease) OR (Chronic Renal Disease) OR (Disease, Chronic Renal) OR (Chronic-insufficienc, Kidney) OR (Chronic insufficienc*, Kidney) OR (Chronic Renal Insufficiency) OR (Chronic Kidney Disease OR Disease*), (Chronic Kidney) OR (Renal Disease*, Chronic) OR (End-Stage Kidney Disease) OR (Disease*, End-Stage Kidney) OR (End Stage Kidney Disease) OR (Kidney Disease*, End-Stage) OR (Chronic Kidney Failure) OR (End-Stage Renal Disease) OR (Disease*, End-Stage Renal) OR (End Stage Renal Disease) OR (Renal Disease*, End-Stage) OR (Renal Disease*, End Stage) OR (Renal Failure, End-Stage) OR (End-Stage Renal Failure) OR (Renal Failure, End Stage) OR (Renal Failure, Chronic) OR (Chronic Renal Failure) OR (Chronic Renal Insufficienc)*OR (Renal Insufficienc*, Chronic) OR (Kidney Insufficienc*, Chronic) OR (Chronic Kidney Insufficienc*) OR (Kidney Insufficience*, Chronic) OR (Chronic Kidney Disease*) OR (Kidney Disease* Chronic) OR (Chronic Renal Disease) OR (Disease*, Chronic Renal) OR (Chronic-

insufficienc*, Kidney) OR (Chronic insufficienc*, Kidney) OR (Chronic Renal Insufficiency) OR (Chronic Kidney Disease*) OR (Disease*, Chronic Kidney) OR (Renal Disease*, Chronic) OR (End-Stage Kidney Disease) OR (Disease*, End-Stage Kidney) OR (End Stage Kidney Disease) OR (Kidney Disease*, End-Stage) OR (Chronic Kidney Failure) OR (End-Stage Renal Disease) OR (Disease*, End-Stage Renal) OR (End Stage Renal Disease) OR (Renal Disease*, End-Stage) OR (Renal Disease*, End Stage) OR (Renal Failure, End-Stage) OR (End-Stage Renal Failure) OR (Renal Failure, End Stage) OR (Renal Failure, Chronic) OR (Chronic Renal Failure)).mp.

AND

((Unknown origin) OR (Uncertain origin) OR (Origin, unknown) OR (Origin, uncertain) OR (Undetermined cause*) OR (Unspecified cause*) OR (Unrevealed cause*) OR (Unrevealed factor*) OR (Unspecified factor*) OR (Factor*,unspecif*) OR (Factor*, undetermined*)OR (Origin, undetermined*) OR (Origin, unspecif*) OR (Origin, unreveal*) OR (Cause*, unreveal*) OR (Cause*, unspecif*) OR (Cause*, undetermined*) OR (Undecided factor) OR (Factor*, undecided*) OR (Cause*, undecided*) OR (Unknown etiolog*) OR (Etiolog*,unknown) OR (Uncertain etiolog*) OR (Unrevealed etiolog*) OR (Undecided etiolog*) OR (Undetermined etiolog*) OR (Etiolog*, uncertain*) OR (Etiolog*, unreveal) OR (Etiolog*, undecide*) OR (Etiolog*, undetermined*) OR (Unknown Associated factor*) OR (Undecided Co-factor*) OR (Unknown Cofactor*) OR (Unknown Factor*, connect*) OR (Unknown Connecting factor*) OR (Undecided Contributing factor*) OR (Undecided Determining factor*) OR (Undecided Contextual factor*) OR (Undecided Relevant factor*) OR (Unknown origin) OR (Uncertain origin) OR (Origin, unknown) OR (Origin, uncertain) OR (Undetermined cause*) OR (Unspecified cause*) OR (Unrevealed cause*) OR (Unrevealed factor*) OR (Unspecified factor*) OR (Factor*,unspecif*) OR (Factor*, undetermined*) OR (Origin, undetermined*) OR (Origin, unspecif*) OR (Origin, unreveal*) OR (Cause*, unreveal*) OR (Cause*, unspecif*) OR (Cause*, undetermined*) OR (Undecided factor) OR (Factor*, undecided*) OR (Cause*, undecided*) OR (Unknown etiolog*) OR (Etiolog*,unknown) OR (Uncertain etiolog*) OR (Unrevealed etiolog*) OR (Undecided etiolog*) OR (Undetermined etiolog*) OR (Etiolog*, uncertain*) OR (Etiolog*, unreveal) OR (Etiolog*, undecide*) OR (Etiolog*, undetermined*) OR (Unknown Associated factor*) OR (Undecided Co-factor*) OR (Unknown Cofactor*) OR (Unknown Factor*, connect*) OR (Unknown Connecting factor*) OR (Undecided Contributing factor*) OR (Undecided Determining factor*) OR (Undecided Contextual factor*) OR (Undecided Relevant factor*)).mp.

EMBASE

‘Chronic Renal Insufficienc*’ OR ‘Renal Insufficienc*, Chronic’ OR ‘Kidney Insufficienc* Chronic’ OR ‘Chronic Kidney Insufficienc*’ OR ‘Kidney Insufficience*, Chronic’ OR ‘Chronic Kidney Disease*’ OR ‘Kidney Disease’ OR ‘Chronic Renal Disease’ OR ‘Disease, Chronic Renal’ OR ‘Chronic-insufficienc, Kidney’ OR ‘Chronic insufficienc*, Kidney’ OR ‘Chronic Renal Insufficienc’ OR ‘Chronic Kidney Disease’ OR ‘Disease*, Chronic Kidney’ OR ‘Renal Disease*, Chronic’ OR ‘End-Stage Kidney Disease’ OR ‘Disease*, End-Stage Kidney’ OR ‘End Stage Kidney Disease’ OR ‘Kidney Disease*, End-Stage’ OR ‘Chronic Kidney Failure’ OR ‘End-Stage Renal Disease’ OR ‘Disease*, End-Stage Renal’ OR ‘End Stage Renal Disease’ OR ‘Renal Disease*, End-Stage’ OR ‘Renal Disease*, End Stage’ OR ‘Renal Failure, End-Stage’ OR ‘End-Stage Renal Failure’ OR ‘Renal Failure, End Stage’ OR ‘Renal Failure, Chronic’ OR ‘Chronic Renal Failure’ OR ‘Chronic Renal Insufficienc*’ OR ‘Renal Insufficienc*, Chronic’ OR ‘Kidney Insufficienc*, Chronic’ OR ‘Chronic Kidney Insufficienc*’ OR ‘Kidney Insufficience*, Chronic’ OR ‘Chronic Kidney Disease*’ OR ‘Kidney Disease* Chronic’ OR ‘Chronic Renal Disease’ OR ‘Disease*, Chronic Renal’ OR ‘Chronic-

insufficienc*, Kidney' OR 'Chronic insufficienc*, Kidney' OR 'Chronic Renal Insufficien*' OR 'Chronic Kidney Disease*' OR 'Disease*, Chronic Kidney' OR 'Renal Disease*, Chronic' OR 'End-Stage Kidney Disease' OR 'Disease*, End-Stage Kidney' OR 'End Stage Kidney Disease' OR 'Kidney Disease*, End-Stage' OR 'Chronic Kidney Failure' OR 'End-Stage Renal Disease' OR 'Disease*, End-Stage Renal' OR 'End Stage Renal Disease' OR 'Renal Disease*, End-Stage' OR 'Renal Disease*, End Stage' OR 'Renal Failure, End-Stage' OR 'End-Stage Renal Failure' OR 'Renal Failure, End Stage' OR 'Renal Failure, Chronic' OR 'Chronic Renal Failure':ab,ti

AND

'Unknown origin' OR 'Uncertain origin' OR 'Origin, unknown' OR 'Origin, uncertain' OR 'Undetermined cause*' OR 'Unspecified cause*' OR 'Unrevealed cause*'OR 'Unrevealed factor*' OR 'Unspecified factor*' OR 'Factor*,unspecif*' OR 'Factor*, undetermined*' OR 'Origin, undetermined*' OR 'Origin, unspecif*' OR 'Origin, unreveal*' OR 'Cause*, unreveal*' OR 'Cause*, unspecif*' OR 'Cause*, undetermined*' OR 'Undecided factor' OR 'Factor*, undecided*' OR 'Cause*, undecided*' OR 'Unknown etiolog*' OR 'Etiolog*,unknown' OR 'Uncertain etiolog*' OR 'Unrevealed etiolog*' OR 'Undecided etiolog*' OR 'Undetermined etiolog*' OR 'Etiolog*, uncertain*' OR 'Etiolog*, unreveal' OR 'Etiolog*, undecide*' OR 'Etiolog*, undetermined*' OR 'Unknown Associated factor*' OR 'Undecided Co-factor*' OR 'Unknown Cofactor*' OR 'Unknown Factor*, connect*' OR 'Unknown Connecting factor*' OR 'Undecided Contributing factor*' OR 'Undecided Determining factor*' OR 'Undecided Contextual factor*' OR 'Undecided Relevant factor*' OR 'Unknown origin' OR 'Uncertain origin' OR 'Origin, unknown' OR 'Origin, uncertain' OR 'Undetermined cause*' OR 'Unspecified cause*' OR 'Unrevealed cause*' OR 'Unrevealed factor*' OR 'Unspecified factor*' OR 'Factor*,unspecif*' OR 'Factor*, undetermined*' OR 'Origin, undetermined*' OR 'Origin, unspecif*' OR 'Origin, unreveal*' OR 'Cause*, unreveal*' OR 'Cause*, unspecif*' OR 'Cause*, undetermined*' OR 'Undecided factor' OR 'Factor*, undecided*' OR 'Cause*, undecided*' OR 'Unknown etiolog*' OR 'Etiolog*,unknown' OR 'Uncertain etiolog*' OR 'Unrevealed etiolog*' OR 'Undecided etiolog*' OR 'Undetermined etiolog*' OR 'Etiolog*, uncertain*' OR 'Etiolog*, unreveal' OR 'Etiolog*, undecide*' OR 'Etiolog*, undetermined*' OR 'Unknown Associated factor*' OR 'Undecided Co-factor*' OR 'Unknown Cofactor*' OR 'Unknown Factor*, connect*' OR 'Unknown Connecting factor*' OR 'Undecided Contributing factor*' OR 'Undecided Determining factor*'OR 'Undecided Contextual factor*' OR 'Undecided Relevant factor*' OR ' Unknown origin' OR 'Uncertain origin' OR 'Origin, unknown' OR 'Origin, uncertain' OR 'Undetermined cause*' OR 'Unspecified cause*' OR 'Unrevealed cause*' OR 'Unrevealed factor*' OR 'Unspecified factor*' OR 'Factor*,unspecif*' OR 'Factor*, undetermined*' OR 'Origin, undetermined*' OR 'Origin, unspecif*' OR 'Origin, unreveal*' OR 'Cause*, unreveal*' OR 'Cause*, unspecif*' OR 'Cause*, undetermined*' OR 'Undecided factor' OR 'Factor*, undecided*' OR 'Cause*, undecided*' OR 'Unknown etiolog*' OR 'Etiolog*,unknown' OR 'Uncertain etiolog*' OR 'Unrevealed etiolog*' OR 'Undecided etiolog*' OR 'Undetermined etiolog*' OR 'Etiolog*, uncertain*' OR 'Etiolog*, unreveal' OR 'Etiolog*, undecide*' OR 'Etiolog*, undetermined*' OR 'Unknown Associated factor*' OR 'Undecided Co-factor*' OR 'Unknown Cofactor*' OR 'Unknown Factor*, connect*' OR 'Unknown Connecting factor*' OR 'Undecided Contributing factor*' OR 'Undecided Determining factor*' OR 'Undecided Contextual factor*' OR 'Undecided Relevant factor*':ab,ti
